# Poor Immunogenicity, Not Vaccine Strain Egg Adaptation, May Explain the Low H3N2 Influenza Vaccine Effectiveness in 2012–2013

**DOI:** 10.1093/cid/ciy097

**Published:** 2018-02-20

**Authors:** Sarah Cobey, Sigrid Gouma, Kaela Parkhouse, Benjamin S Chambers, Hildegund C Ertl, Kenneth E Schmader, Rebecca A Halpin, Xudong Lin, Timothy B Stockwell, Suman R Das, Emily Landon, Vera Tesic, Ilan Youngster, Benjamin A Pinsky, David E Wentworth, Scott E Hensley, Yonatan H Grad

**Affiliations:** 1 Department of Ecology & Evolution, University of Chicago, Illinois; 2 Department of Microbiology, Perelman School of Medicine, University of Pennsylvania, Philadelphia; 3 Division of Geriatrics, Duke University Medical Center, North Carolina; 13 Geriatric Research, Education, and Clinical Center, Durham VA Medical Center, North Carolina; 4 Department of Infectious Disease, J. Craig Venter Institute, Rockville, Maryland; 5 Department of Medicine, University of Chicago, Illinois; 6 Department of Pathology, University of Chicago, Illinois; 7 Division of Pediatrics and the Center for Microbiome Research, Assaf Harofeh Medical Center, Tel Aviv University, Israel; 8 Division of Infectious Diseases, Boston Children’s Hospital, Massachusetts; 9 Department of Pathology, Stanford University School of Medicine, California; 10 Division of Infectious Diseases and Geographic Medicine, Department of Medicine, Stanford University School of Medicine, California; 11 Department of Immunology and Infectious Diseases, Harvard TH Chan School of Public Health, Boston, Massachusetts; 12 Division of Infectious Diseases, Brigham and Women’s Hospital, Harvard Medical School, Boston, Massachusetts

**Keywords:** influenza, vaccine effectiveness, egg adaptation, genome sequencing, vaccine immunogenicity

## Abstract

**Background:**

Influenza vaccination aims to prevent infection by influenza virus and reduce associated morbidity and mortality; however, vaccine effectiveness (VE) can be modest, especially for subtype A(H3N2). Low VE has been attributed to mismatches between the vaccine and circulating influenza strains and to the vaccine’s elicitation of protective immunity in only a subset of the population. The low H3N2 VE in the 2012–2013 season was attributed to egg-adaptive mutations that created antigenic mismatch between the actual vaccine strain (IVR-165) and both the intended vaccine strain (A/Victoria/361/2011) and the predominant circulating strains (clades 3C.2 and 3C.3).

**Methods:**

We investigated the basis of low VE in 2012–2013 by determining whether vaccinated and unvaccinated individuals were infected by different viral strains and by assessing the serologic responses to IVR-165, A/Victoria/361/2011, and 3C.2 and 3C.3 strains in an adult cohort before and after vaccination.

**Results:**

We found no significant genetic differences between the strains that infected vaccinated and unvaccinated individuals. Vaccination increased titers to A/Victoria/361/2011 and 3C.2 and 3C.3 representative strains as much as to IVR-165. These results are consistent with the hypothesis that vaccination boosted cross-reactive immune responses instead of specific responses against unique vaccine epitopes. Only approximately one-third of the cohort achieved a ≥4-fold increase in titer.

**Conclusions:**

In contrast to analyses based on ferret studies, low H3N2 VE in 2012–2013 in adults does not appear to be due to egg adaptation of the vaccine strain. Instead, low VE might have been caused by low vaccine immunogenicity in a subset of the population.

## Teaser

Low H3N2 vaccine effectiveness in 2012–2013 appears attributable to low vaccine immunogenicity in a subset of adults, and not the induction of a narrow, specific response to the egg-adapted strain suggested by previous experiments in immunologically naive ferrets.

Vaccination is one of the most important public health interventions to mitigate the annual threat of influenza. Each season, an estimated 4% of individuals aged <65 years seek outpatient care for influenza-related symptoms, and 12000–56000 people die from influenza infection in the United States [1, 2]. Vaccination in the US population has been increasing, with coverage in the 2016–2017 season ranging from 76% in those aged 6–23 months to 33.6% in individuals aged 18 to 49 years and 65.3% in individuals aged ≥65 years [3]. However, the vaccine effectiveness (VE) continues to be modest, particularly against the A(H3N2) viruses, with a recent metaanalysis reporting pooled VE of 33% (95% confidence interval [CI], 26–39) for A(H3N2), 61% (57–65) for A(H1N1pdm09), and 54% (46–61) for B [4].

What explains this modest effectiveness, particularly for the H3N2 subtype? A common explanation is that the vaccine and circulating strains are antigenically different, implying that vaccination protects against the vaccine strain but is less protective against antigenically distinct circulating strains. Metaanalyses have indicated that VE to antigenically drifted viruses is worse than to matched viruses [4, 5]. Mismatches can arise from inaccurate prediction of the future strains that will dominate a season and/or the significant antigenic diversity of cocirculating strains [6–8].

Antigenic mismatches can also be caused by mutations that arise as human vaccine strains are propagated in fertilized chicken eggs for large-scale vaccine production [9, 10]. For example, the egg-adapted 2016–2017 H3N2 vaccine strain was antigenically distinct compared with circulating H3N2 strains due to a single hemagglutinin (HA) mutation that arose as the vaccine strain was adapted to replicate efficiently in eggs [10]. Many humans who received the 2016–2017 vaccine produced antibodies that recognized the egg-grown H3N2 vaccine strain but not circulating H3N2 strains [10]. It has been proposed that egg adaptations also contributed to lowered VE during the H3N2-dominated 2012–2013 season [11], when influenza VE was estimated to be 39% (95% CI, 29–47) [12]. The 2012–2013 egg-adapted A/Victoria/361/2011-IVR-165 (IVR-165) vaccine strain differed from the cell-cultured A/Victoria/361/2011 (Vic/361) strain at several residues in exposed regions of the HA head near the receptor-binding site (H156Q, G186V, S219Y) [11].

Low VE during the 2012–2013 season might also have arisen from poor vaccine-induced protection in some hosts [13, 14]. Differences in VE by age [4, 12] have led to speculation that age-associated differences in infection and vaccination history [15, 16] affect protection [17, 18]. Notably, naive ferrets infected with IVR-165 developed titers that were 16- to 32-fold lower against Vic/361 compared with IVR-165, but naive ferrets infected with Vic/361 developed similar titers to both strains [11, 19]. People previously uninfected with H3N2 viruses who were immunized with the IVR-165 vaccine strain might have similarly distinct responses that neutralized the egg-adapted IVR-165 strain more efficiently than circulating H3N2 strains. It is unclear, however, if the IVR-165 vaccine strain would elicit this type of response in human adults who have had prior H3N2 exposures, as prior exposure might expand the range of responses to a vaccine [20–22].

To investigate the basis of the low VE against H3N2 in 2012–2013, we first determined whether vaccinated and unvaccinated individuals were infected by different viral strains. If vaccinated people were infected with antigenically distinct strains, then low VE may be partially attributable to mismatch of some kind. We then evaluated the serologic responses to Vic/361, IVR-165, and representative circulating strains in an adult cohort before and after vaccination. If low VE arose from mismatch due to egg adaptations in IVR-165, we expected to see a robust response to IVR-165 but not to Vic/361 or circulating wild-type strains.

## METHODS

### Virus Specimens

The dataset comprises influenza A(H3N2) samples from 2012–2013 collected for this study and sequences collated from online databases. The samples collected for this study were from clinical specimens identified as positive for influenza in the microbiology laboratories in 3 hospitals in Boston, Massachusetts (Brigham and Women’s Hospital, Massachusetts General Hospital, and Boston Children’s Hospital), 1 in Chicago, Illinois (University of Chicago Hospital), and 1 in Santa Clara, California (Stanford University Hospital). The cycle threshold value and the type and subtype of influenza were recorded, when available. Patient demographics (including gender and age) and vaccination status were documented based on review of medical records. The study was performed with institutional review board (IRB) approval from each participating institution (Partners Healthcare IRB protocol 2013-P-000097/1; Boston Children’s Hospital IRB protocol IRB-2013-P00007299; University of Chicago IRB protocol 13–0149; Stanford IRB protocol 27044). Sequences were also obtained from the National Center for Biotechnology Information influenza virus database (https://www.ncbi.nlm.nih.gov/genomes/FLU/Database/nph-select.cgi?go=database). The query specified full-length HA H3N2 specimens from the United States from 1 September 2012 to 1 June 2013. To these sequences, we added 23 partial HA sequences from Dinis et al [23] for which the vaccination status of the infected individual was reported. We annotated the genome sequences by date and location of isolation, when available, as well as the vaccination status of the infected individuals [12, 23].

### Sequencing of the Influenza Genomes

The complete genomes of the influenza A viruses collected were sequenced as part of the National Institutes of Health, National Institute of Allergy and Infectious Diseases, sponsored Influenza Genome Sequencing Project. Viral RNA was isolated using the ZR 96 Viral RNA kit (Zymo Research Corporation, Irvine, California). The influenza A genomic RNA segments were simultaneously amplified from 3 µL of purified RNA using a multisegment reverse transcription polymerase chain reaction (M-RTPCR) strategy [24, 25]. The M-RTPCR amplicons were used as templates for Nextera Library construction, and libraries were sequenced using the MiSeq v2 platform (Illumina, Inc., San Diego, California). The sequence reads from the MiSeq were sorted by barcode and trimmed, and noninfluenza sequences were removed. The next generation sequencing (NGS) reads were then mapped to the best matching reference virus using the clc_ref_assemble_long program. At loci where NGS platforms agreed on a variation (compared with the reference sequence), the reference sequence was updated to reflect the difference. A final mapping of all next-generation sequences to the updated reference sequences was then performed.

### Phylogenetic Analysis

HA sequences from the full set of H3N2 influenza strains from 2012–2013 were aligned using MUSCLE [26], and a maximum likelihood tree was generated using RAxML [27] with default options. Phylogenies and metadata were visualized in Phandango [28]. To determine the association of vaccination status and phylogeny, the Fitz and Purvis *D* statistic was calculated in R using phylo.d from the caper package [29] using a maximum likelihood tree comprising the isolates with known vaccination status.

### Human Participants and Sera

As part of a cohort study [30–32], blood was collected from 61 adult participants from the Durham–Raleigh–Chapel Hill, North Carolina, area at the Duke Clinical Research Unit, Duke University Medical Center, Durham, North Carolina. Twenty-eight participants were aged 30–40 years, and 33 were aged 65–87 years. For some individuals, the volume of sera available limited the number of hemagglutination inhibition (HAI) assays that could be performed. Self-reported vaccination history was also available, and vaccination in 2011–2012 could be confirmed in participants who had enrolled in the study the previous year. Most individuals had been vaccinated at least once since 2009 before receipt of the 2012–2013 vaccine.

### Ferret Sera

Sera isolated from 6 ferrets intranasally inoculated with a tissue-culture grown preparation of A/Victoria/361/2012 was obtained from the International Reagent Resource (FR-1079). Sera isolated from 1 ferret intranasally inoculated with the A/Victoria/361/2012 IVR-165 strain was kindly provided by Dr Xiyan Xu (Centers for Disease Control and Prevention).

### Virus Propagation and Characterization

We used reverse genetics to produce viruses that expressed HA from Vic/361, IVR-165, and clades 3C.2 and 3C.3 representatives (GenBank accessions CY171703.1 and CY170119.1, respectively). These viruses possessed the same Vic/361 NA and 6 internal protein coding genes from the A/Puerto Rico/8/1934 (H1N1) virus. The HAs of Vic/361 and IVR-165 differed at residues 156 (H156Q), 186 (G186V), and 219 (S219Y). We propagated viruses for 2 days using MDCK-SIAT1 cells (Madin-Darby canine kidney cells stably transfected with human 2,6-sialyltransferase), and we used standard Sanger sequencing to verify that other HA mutations did not predominate after virus propagation.

### Hemagglutination Inhibition Assays

Sera were pretreated with receptor-destroying enzyme (Key Scientific Products, Inc., Stamford, Texas) and heat inactivated for 30 minutes at 55°C. Pretreated sera were incubated with a guinea pig erythrocyte solution (10% vol/vol) to remove serum components that bind erythrocytes. HAI titrations were performed in 96-well V-bottom plates (Greiner, Monroe, North Carolina). Sera were serially diluted 2-fold and added to 4 agglutinating doses of virus in a total volume of 100 µL. Guinea pig erythrocyte solution (12.5 µL; 2.5% vol/vol; Lampire, Pipersville, Pennsylvania) was added to sera–virus mixtures. Contemporary H3N2 viruses often acquire NA mutations during in vitro propagation, and these mutations can confound HAI assays [33]. To verify that agglutination of guinea pig erythrocytes of our viral preps was HA mediated, we completed all HAI assays in the presence of 20 nM oseltamivir, a compound that binds in the sialic acid binding site of NA. Agglutination was read out after incubating erythrocytes for 1 hour at room temperature followed by tilting of the plates for 1 minute. HAI titers were expressed as the inverse of the highest dilution that inhibited 4 agglutinating doses of guinea pig erythrocytes, measured by a full “tear drop pattern.” When a well showed a tear drop pattern that was not full, the HAI titer was considered to be 0.75 times the inverse of this dilution. For the analysis, HAI titers <20 were assigned values of 10. Titers to IVR-165 were similar regardless of whether the strain was grown in MDCK-SIAT1 cells or eggs (Supplementary Figure 1), and the default analyses used titers to cell-grown IVR-165. There was also good agreement between 2 replicate titer measurements (Supplementary Figure 2), and titers from the first replicate were used by default.

## RESULTS

### Similar H3N2 Strains Infected Vaccinated and Unvaccinated People

**Figure 1.**
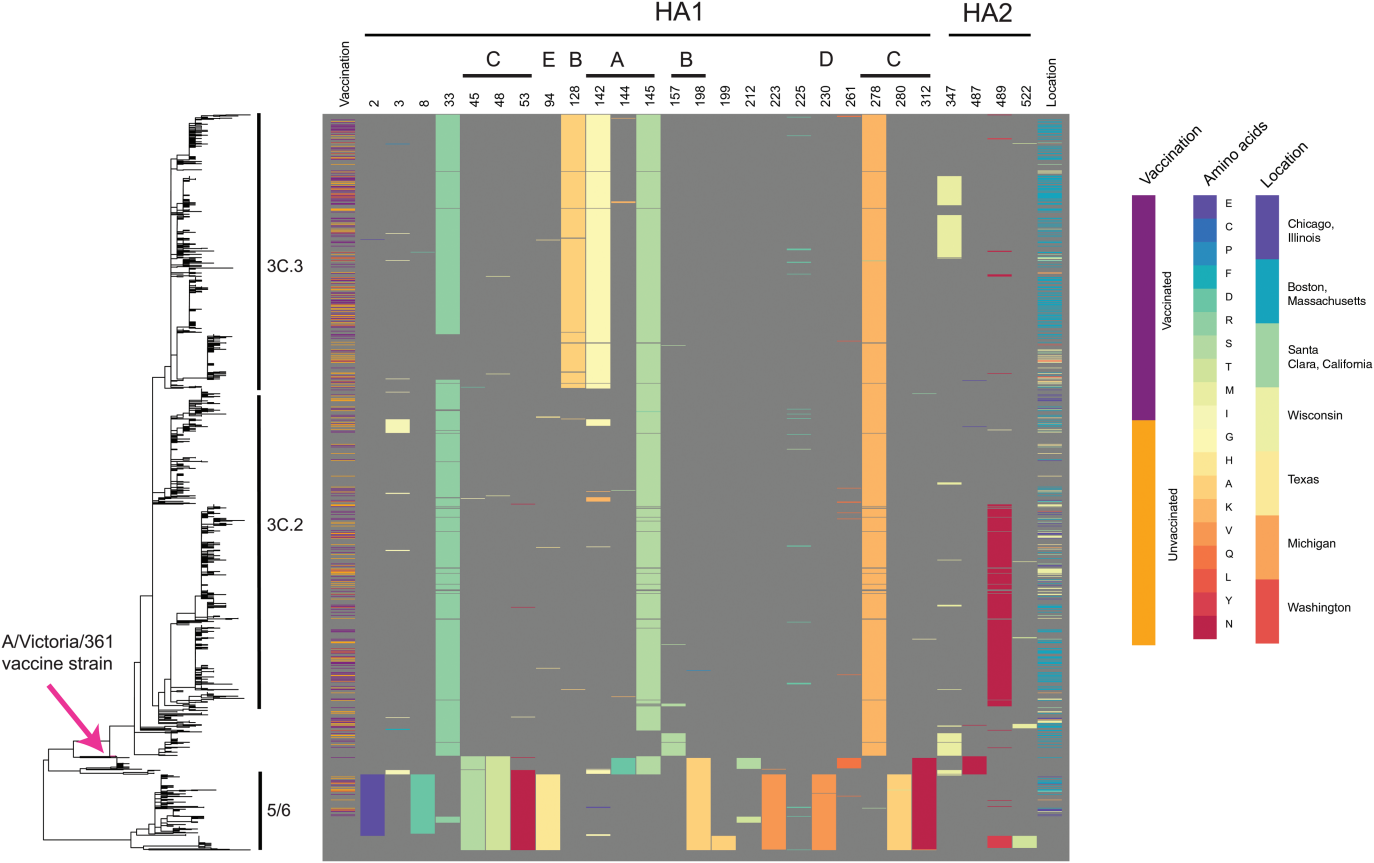
Maximum likelihood phylogeny of hemagglutinin (HA) sequences from 1339 influenza A(H3N2) isolates collected from North America during the 2012–2013 influenza season. Vaccination status of the individuals from whom the isolates were collected is noted (purple = vaccinated; orange = unvaccinated; blank = unknown vaccination status). Amino acid sites in which 20 or more of the 1339 specimens differed from the vaccine strain IVR-165 are noted, with the amino acids colored according to the key and annotated according to their location in HA1, HA2, and predicted epitope sites (A–E). The location from which the isolates were collected is color coded according to the key. Abbreviation: HA, hemagglutinin.

To determine whether vaccinated and unvaccinated individuals were infected with different viral strains, we collated 423 influenza A(H3N2) sequences from individuals with known vaccination status infected during the 2012–2013 season in the United States. This dataset comprised 316 specimens sequenced for the purposes of this study and 2 published collections [12, 23] (Supplementary Table 1). Analysis of the HA sequences revealed no phylogenetic clustering by vaccination status (Figure 1), with the Fritz and Purvis [34] estimated *D* statistic of 0.93, consistent with a random association between vaccination status and the phylogeny. This is in keeping with a previous observation in which a smaller dataset was used [23].

Meaningful differences between the strains that infect vaccinated and unvaccinated individuals may be focused in sites within HA that impact antigenicity and avidity [35, 36]. To identify variable HA amino acid sites from the 2012–2013 influenza season, we assembled a dataset of 1339 complete HA sequences from the United States from 2012–2013 (Supplementary Table 2), including the set of 423 sequences described above, and determined the amino acid sites in which 20 or more specimens differed from the vaccine strain IVR-165. We identified 23 variable amino acid sites in HA1 and 4 variable sites in HA2 (Figure 1) using these criteria. We then determined the haplotypes of these 27 sites in individuals with known vaccination status, yielding 33 haplotypes. The frequency with which these haplotypes appeared ranged from rare (17 haplotypes appeared only once) to common (122 of the 423 samples shared the same haplotype at these variable sites; Supplementary Table 3). For the 3 most abundant haplotypes, we determined whether the samples from vaccinated individuals were overrepresented. A haplotype that contained HA2 V18M trended toward significance (Bonferroni corrected *p* value of 0.1). However, 55 of 59 isolates with this haplotype were from Boston, which had a high ratio of vaccinated-to-unvaccinated individuals (1.7:1) compared with other sites (0.8:1), indicating even less significance. Interestingly, the haplotype analysis revealed evidence of convergent evolution, with identical amino acid variants appearing in multiple clades (eg, N225D appeared in clades 3C.3, 3C.2, and 5/6). Together, these data suggest that there are no statistically significant differences in haplotypes between vaccination groups in a season that showed the emergence of new HA lineages (3C.2 and 3C.3). Rather, both vaccinated and unvaccinated individuals were infected with antigenically diverged clades.

### Vaccination Boosts Serum Antibody Responses to Vaccine, Wild-Type, and Circulating Strains Equally

**Figure 2.**
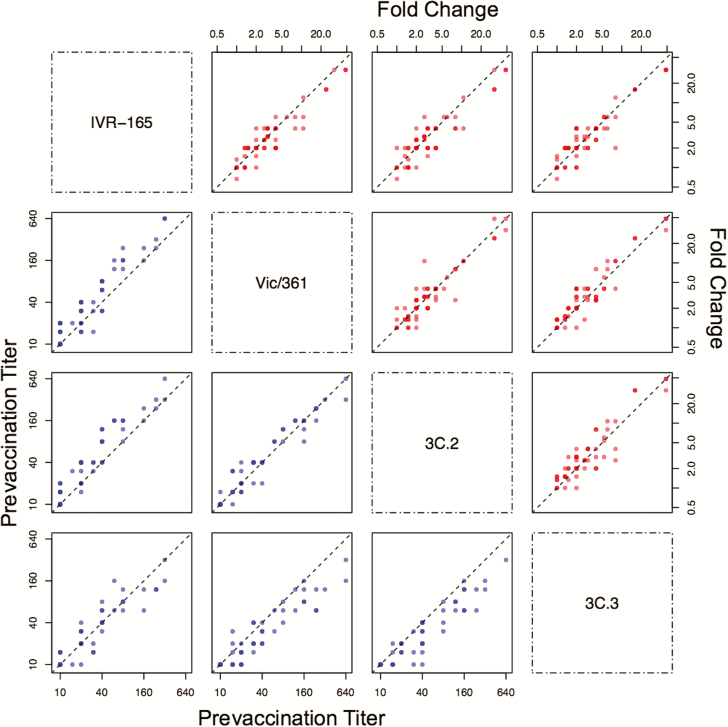
Correlations between prevaccination titers (lower left panels, blue) and fold changes in titers (upper right panels, red). Fold changes are defined as the post-vaccination titer divided by the prevaccination titer. Points are semitranslucent; darker points represent multiple individuals. The dotted black line shows the diagonal (1:1) curve. On each plot, the *x*-axis corresponds to the strain for that column, and the *y*-axis corresponds to the strain for that row.

Because vaccinated people were infected with similar strains as unvaccinated people, antibody responses induced by the vaccine might have been very weak or they might have recognized epitopes present in the vaccine strain but absent in circulating strains. It has been proposed that egg adaptations in the IVR-165 vaccine strain contributed to low VE during the 2012–2013 season [11]. Consistent with previous reports [11, 19], we found that naive ferrets infected with IVR-165 generated antibodies that reacted poorly with the wild-type Vic/361 strain (Supplementary Table 4). However, we found that vaccinated adult humans produced antibodies that recognized both Vic/361 and IVR-165, as well as strains from the dominant wild-type clades 3C.2 and 3C.3, which circulated that season (Figure 2 and Supplementary Figure 3). For these experiments, we completed HAI assays using sera from adults before and after immunization with IVR-165.

Adults’ prevaccination titers were similar to all strains. Before vaccination, at least one-third of 57 adults had titers ≥40 to Vic/361 (22 adults), IVR-165 (19 adults), a representative strain of clade 3C.2 (27 adults), and a representative strain of clade 3C.3 (19 adults; Figure 2). The prevaccination titers were correlated; for example, 16 of 22 individuals with titers ≥40 to Vic/361 had titers ≥40 to the other 3 strains.

After vaccination with IVR-165, titers to IVR-165 increased in 86% (48 of 56) of individuals and increased by at least 4-fold in 36% (20 of 56). Strikingly, despite variation in individual’s responses to IVR-165, there was a strong linear correlation between the log_2_-fold changes in titer to IVR-165 and Vic/361 (Pearson correlation = 0.93 [CI, 0.88, 0.96], *P* < 10^–15^; Figure 2), including when the comparison to traditionally defined responders was restricted (Supplementary Figure 4). The fold change distributions were not significantly different, although there was a trend toward greater boosting to Vic/361 than IVR-165 (paired Wilcoxon signed rank test, approximate *P* value = .07). There were similarly strong linear correlations between individuals ’ antibody increases to IVR-165 and circulating clades (Figure 2); again, the fold changes did not significantly differ (with 3C.3, approximate *P* value = .52) or were slightly higher for the nonvaccine strain (for 3C.2, approximate *P* value = .11). Thus, regardless of strength, responses to the vaccine strain induced almost the same magnitude change that was seen in response to the wild-type and circulating strains, suggesting that the vaccine induced antibodies that cross-reacted with the other strains and effectively only antibodies that cross-reacted with the other strains. The strong correlations between prevaccine titers (Figure 2) suggest that these antibodies predated exposure to IVR-165.

Adults did not respond like naive ferrets, which developed 4-fold greater responses to IVR-165 than to Vic/361 after vaccination with IVR-165 (Supplementary Table 4); no humans had fold changes to IVR-165 that were more than 2-fold greater than changes to Vic/361. Of the 16 individuals who started with undetectable titers (<15) to both IVR-165 and Vic/361 and who were thus possibly “naive” to these strains, all but 1 had titers to Vic/361 that were at least as high as those to the vaccine strain.

## DISCUSSION

Influenza VE measures how well a vaccine protects against diverse influenza viruses that circulate during a season. Given the high morbidity related to H3N2 infections [37, 38], there is a vital need to understand why this subtype has lower VE in order to guide improvement of influenza vaccines. The 2 general explanations for why the vaccine fails to achieve high effectiveness are poor vaccine match, where “match” refers to the antigenic similarity between the vaccine and circulating influenza strains [4, 5], and heterogeneous responses to influenza vaccination, such that the vaccine elicits protective immunity in only some people [13, 14].

For the 2012–2013 influenza season, the H3N2 VE was estimated to be 39% [12]. Antigenic data from ferrets support the contention that vaccination with the egg-adapted variants such as IVR-165 yield antibodies that react poorly with the intended Vic/361 vaccine strain. Under this model, the observed VE comes from mismatch with Vic/361, which presumably correlates with the degree of mismatch with circulating strains. In theory, the VE would have been higher without the egg-adaptive mutations, although some mismatch between Vic/361 and circulating strains might have led to reduced effectiveness.

In contrast with the ferret data, we found that human adult responses to Vic/361, 3C.2, 3C.3, and IVR-165 strains before and after vaccination with IVR-165 were similar. We speculate that the differences between the specificity of antibodies elicited in ferrets and humans in our study are due to prior H3N2 exposures in humans. There is extensive evidence that B-cell responses to influenza strains evolve from preexisting responses [16, 17, 21, 39–41]. The immunological concept of “original antigenic sin” in fact arose partly from observations that influenza vaccination boosted responses that cross-react with previously encountered strains, leading to different patterns of vaccine-induced cross-reactivity by cohort [20–22]. If preexisting responses are boosted exclusively, then adults may have failed to detect the antigenic novelty of Vic/361, even without the egg adaptations. In contrast, egg adaptations may have been more recognizable in children due to their shorter exposure history. Differences in the specific epitopes people target could have important consequences for protection and vaccine effectiveness [16, 40, 42].

Interaction between the type of egg-adaptive mutation and immune history may explain why egg adaptation apparently played a large role during the 2016–2017 season [10] but not the 2012–2013 season. Notably, the 2016–2017 H3N2 vaccine strain possessed an egg-adapted HA mutation (T160K) that resulted in loss of a glycosylation site. Changes in HA glycosylation sites can have large antigenic impact. The 2012–2013 H3N2 egg-adapted vaccine possessed a mutation at HA residue 156 (H156Q), which is in the middle of an important antigenic site [36], but did not affect any of the glycosylation motifs on the HA. Prior exposures might have amplified the effects of these differences. Before the 2016–2017 season, many adults had already been exposed to H3N2 viruses that possessed HA with K160, and the egg-adapted 2016–2017 vaccine likely recalled memory B cells to this site. Adults thus boosted titers to the vaccine strain but not circulating strains, which were glycosylated at that site [10]. In contrast, before the 2012–2013 season, few adults had been exposed to H3N2 viruses that possessed HA with Q156, because these viruses had not circulated widely. Therefore, most adults probably did not have memory B cells against the egg-adapted HA epitope on the 2012–2013 H3N2 vaccine strain prior to the 2012 season. They thus boosted responses to conserved epitopes that were also present on circulating strains.

We also observed heterogeneity in the extent of response to vaccination, with some individuals having persistently low HAI titers to the vaccine strain despite vaccination. There is accumulating evidence that increased influenza exposure and older age, which are partly confounded variables [43], are associated with lower responses to vaccination. However, it is unclear why some individuals have lower titers than others after seemingly similar exposure histories.

There were no significant differences at the haplotype level between the strains that infected vaccinated vs unvaccinated people. Although this test lacks power, since vaccine recipients who became infected might have been those with a poor response to the vaccine, the results are consistent with the hypothesis that vaccination served merely to boost preexisting responses in a subset of the population.

Together, these data suggest that the low H3N2 VE in the 2012–2013 season was not primarily attributable to egg-adaptation mutations in the H3N2 vaccine strain. The widely variable, and still inexplicable, immunological responses to the vaccine in the vaccinated population might thus explain the low VE.

## Supplementary Data

Supplementary materials are available at *Clinical Infectious Diseases* online. Consisting of data provided by the authors to benefit the reader, the posted materials are not copyedited and are the sole responsibility of the authors, so questions or comments should be addressed to the corresponding author.

Supplemental Table S1

Supplemental Table S2

Supplemental Table S3

Supplemental Table S4

Supplemental Figure S1

Supplemental Figure S2

Supplemental Figure S3

Supplemental Figure S4

Supplemental Figure Legends

## Notes

***Acknowledgments.*** The authors thank Danuta Skowronski for comments on the manuscript, Kangchon Kim for technical consultation on H3N2 evolution, Nadia Fedorova and Susmita Shrivastava for technical expertise in support of the viral assembly and submission pipeline for this project, and Rebecca Zaffini for assistance with specimen collection. The data for this manuscript were generated while D. E. W. was employed at JCVI.

***Data availability.*** All sequences are available in GenBank; accession numbers are provided in the Supplementary Material.

***Disclaimer.*** The opinions expressed in this article are the authors and do not reflect the views of the Centers for Disease Control, the Department of Health and Human Services, or the US government.

***Financial support.*** This work was supported in part with federal funds from the National Institutes of Health– National Institute of Allergy and Infectious Diseases (grants 1R01AI113047 and 1R01AI108686 to S. E. H.; DP2AI117921 to S. C.; and CEIRS HHSN272201400005C to S. E. H. and S. C.), Department of Health and Human Services (contract HHSN272200900007C), the Burroughs Wellcome Fund (Investigators in the Pathogenesis of Infectious Disease Award to S. E. H.), the Smith Family Foundation (to Y.H.G.), and the Doris Duke Charitable Foundation (Clinical Scientist Development Award to Y. H. G.). We appreciate technical assistance on serological assays from Theresa Eilola and Igor Dombrovsky.

***Potential conflicts of interest.*** S. H. E. reports personal fees from Lumen Bioscience and Merck unrelated to this project, and Y. H. G. reports personal fees from G. S. K. unrelated to this project.
