# Supplementary material for: Poor Immunogenicity, Not Vaccine Strain Egg Adaptation, May Explain the Low H3N2 Influenza Vaccine Effectiveness in 2012–2013
Source: Clin Infect Dis. 2018 Feb 20;67(3):327–33. doi: 10.1093/cid/ciy097 (PMC6051447; doi:10.1093/cid/ciy097)
Supplement: Supplemental Table S4 [file ciy097_suppl_supplemental_table_s4.docx]

**Supplemental Table S4. Ferret antisera titers to IVR-165 (both grown in eggs and in MDCK-SIAL1) and wild type (WT), example 3C.2 and 3C.3 strains after vaccination to IVR-165 or WT. The titers from multiple experiments are reported, with ‘n’ indicating the number of experiments with the reported titer. E.g., ‘640 (n=6)’ indicates 6 separate experiments with a titer of 640.**

|  | A/Vic/361/11 HA IVR-165 grown in eggs | A/Vic/361/11 HA IVR-165 grown in MDCK-SIAL1 | A/Vic/361/11 HA WT | GenBank CY171703.1 3C.2 | GenBank CY170119.1 3C.3 |
| --- | --- | --- | --- | --- | --- |
| Ferret antisera WT | 160 (n=2) | 160 (n=6) | 960 (n=3); 480 (n=2); 640 (n=1) | 1280 (n=1); 640 (n=2); 480 (n=2) | 480 (n=1); 320 (n=2); 240 (n=2) |
| Ferret antisera IVR-165 | 2560 (n=1); 1280 (n=1) | 1920 (n=1); 1280 (n=5) | 480 (n=3); 320 (n=3) | 640 (n=1); 320 (n=4) | 240 (n=1); 160 (n=4) |
