# Supplementary figures and images for: Poor Immunogenicity, Not Vaccine Strain Egg Adaptation, May Explain the Low H3N2 Influenza Vaccine Effectiveness in 2012–2013
Source: Clin Infect Dis. 2018 Feb 20;67(3):327–33. doi: 10.1093/cid/ciy097 (PMC6051447; doi:10.1093/cid/ciy097)

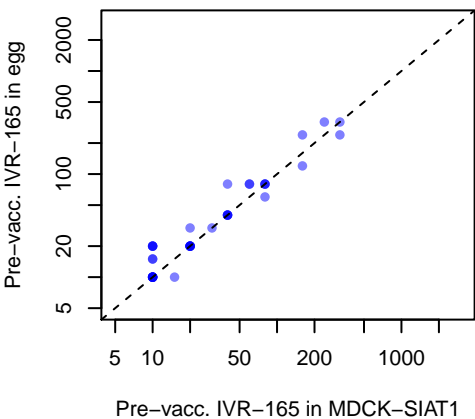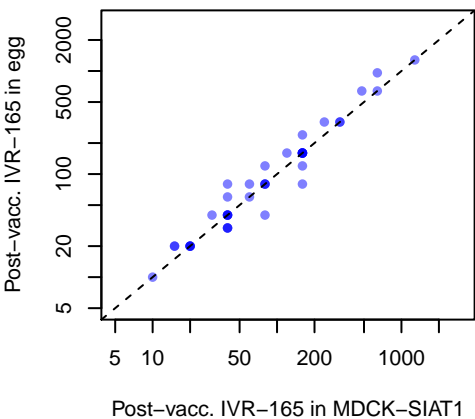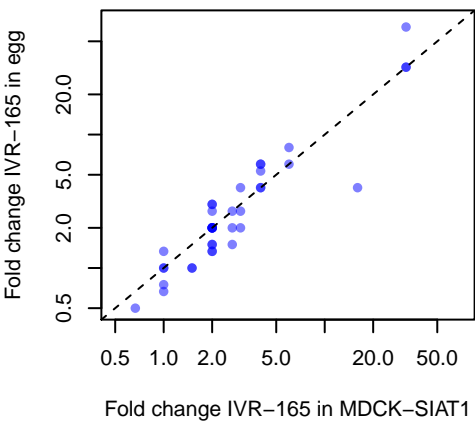

Supplement: Supplemental Figure S1 [file ciy097_suppl_supplemental_figure_s1.pdf]

Fold change IVR-165

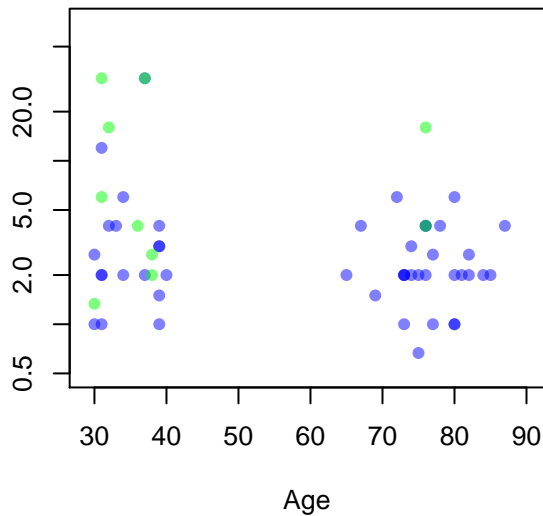

Fold change Vic/361

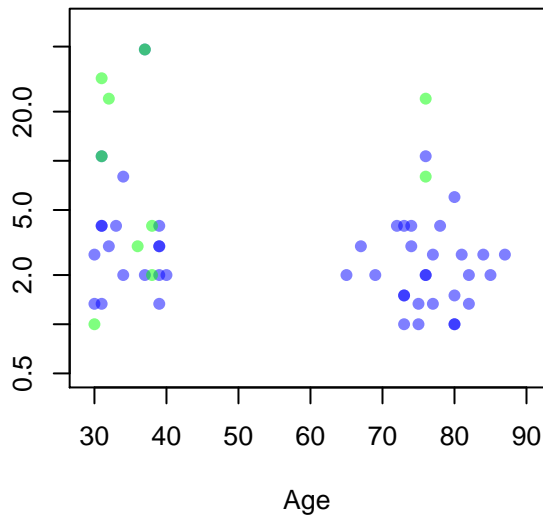

Fold change 3C.2

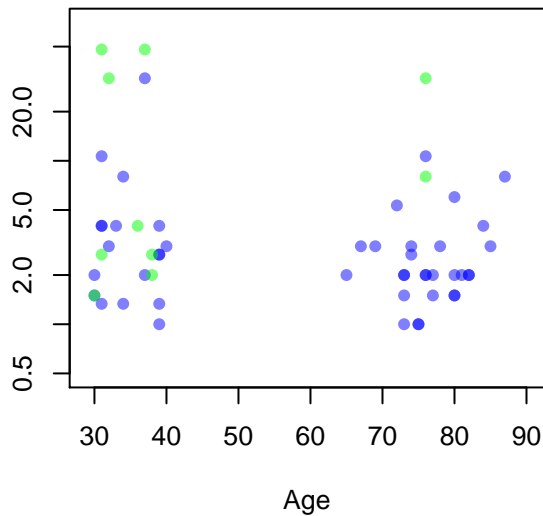

Fold change 3C.3

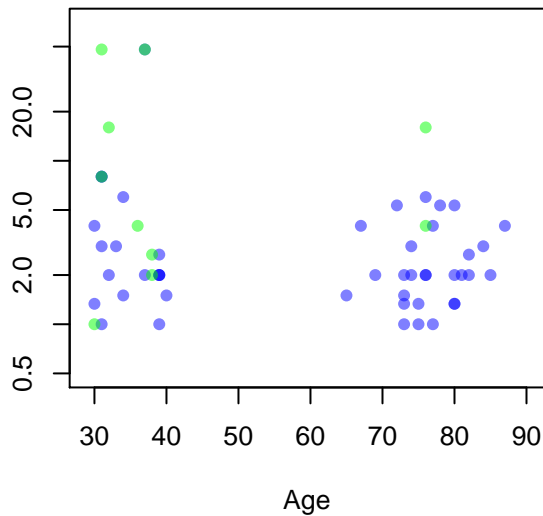

Supplement: Supplemental Figure S3 [file ciy097_suppl_supplemental_figure_s3.pdf]

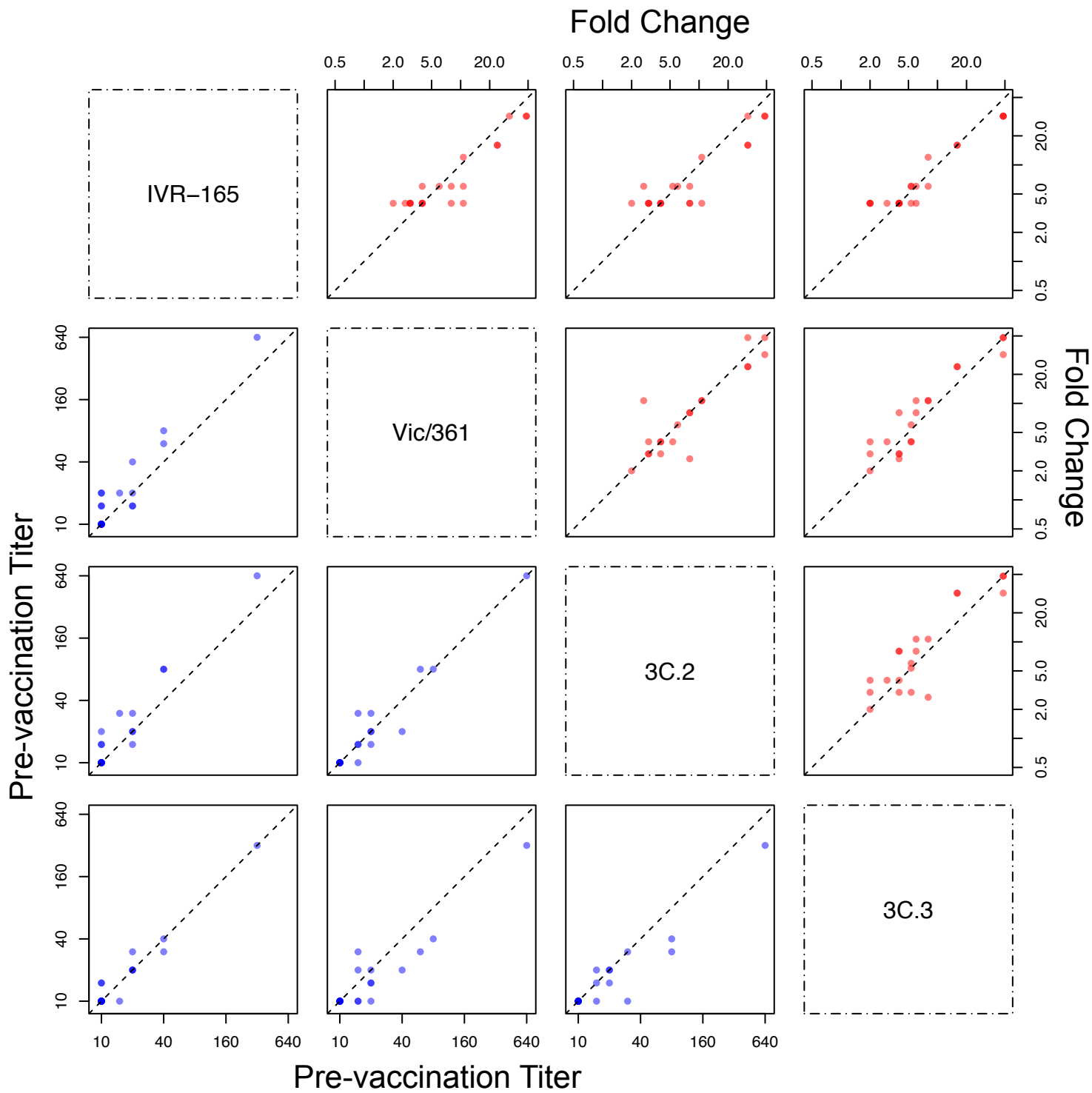

Supplement: Supplemental Figure S4 [file ciy097_suppl_supplemental_figure_s4.pdf]
