# Supplementary material for: Poor Immunogenicity, Not Vaccine Strain Egg Adaptation, May Explain the Low H3N2 Influenza Vaccine Effectiveness in 2012–2013
Source: Clin Infect Dis. 2018 Feb 20;67(3):327–33. doi: 10.1093/cid/ciy097 (PMC6051447; doi:10.1093/cid/ciy097)
Supplement: Supplemental Figure S2 [file ciy097_suppl_supplemental_figure_s2.pdf]

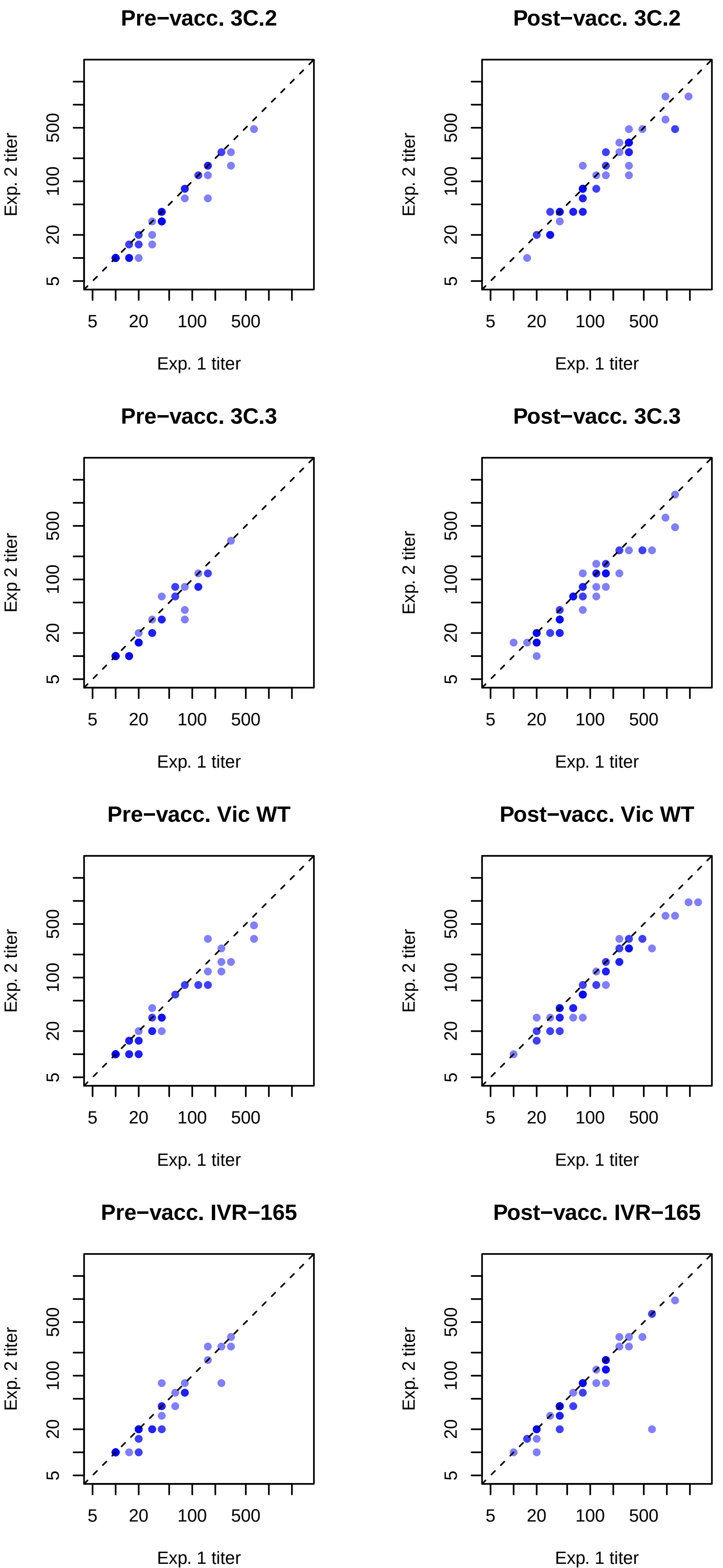
Pre−vacc. 3C.2
Post−vacc. 3C.2
Pre−vacc. 3C.3
Post−vacc. 3C.3
Pre−vacc. Vic WT
Post−vacc. Vic WT
Pre−vacc. IVR−165
Post−vacc. IVR−165
Exp. 2 titer
Exp 2 titer
Exp. 1 titer
5
20
100
500
