# Supplementary material for: Poor Immunogenicity, Not Vaccine Strain Egg Adaptation, May Explain the Low H3N2 Influenza Vaccine Effectiveness in 2012–2013
Source: Clin Infect Dis. 2018 Feb 20;67(3):327–33. doi: 10.1093/cid/ciy097 (PMC6051447; doi:10.1093/cid/ciy097)
Supplement: Supplemental Figure Legends [file ciy097_suppl_supplemental_figure_legends.docx]

**Supplemental figure legends:**

**Supplemental Figure S1**. Correlations between titers to IVR-165 grown in MDCK-SIAT1 cells and eggs in pre-vaccination and post-vaccination visits, and correlations between the fold changes (the ratio of the post-vaccination to the pre-vaccination titer). Points are semi-translucent; darker points represent multiple individuals.

**Supplemental Figure S2.** Correlations between HAI measurements of the same sera on two different dates. All HAI assays from each experiment were performed on the same date. Experiment 1 was performed before experiment 2. Points are semi-translucent; darker points represent multiple individuals

**Supplemental Figure S3**. Fold changes, denoting the ratio of post-vaccination to pre-vaccination titers, in individuals as a function of age and vaccination history. Individuals who had previously not been vaccinated since 2009 are in green, and those who had been vaccinated are in blue. Points are semi-translucent; darker points represent multiple individuals.

**Supplementary Figure S4**. Pre-vaccination titers and fold changes in vaccine responders, defined as the subpopulation with a ≥4-fold increase to IVR-165 (in MDCK-SIAT1 cells), as in Fig. 2.
